# Vitamin B_12_ and/or folic acid supplementation on linear growth: a 6-year follow-up study of a randomised controlled trial in early childhood in North India

**DOI:** 10.1017/S0007114522002343

**Published:** 2023-04-14

**Authors:** Sunita Taneja, Ranadip Chowdhury, Ingrid Kvestad, Nita Bhandari, Tor A. Strand

**Affiliations:** 1 Centre for Health Research and Development, Society for Applied Studies, New Delhi, India; 2 Regional Centre for Child and Youth Mental Health and Child Welfare, West, NORCE Norwegian Research Centre, Bergen, Norway; 3 Department of Research, Innlandet Hospital Trust, Lillehammer, Norway

**Keywords:** Vitamin B_12_, Folic acid, Randomised controlled trial, Long-term effects, Linear growth, Childhood, India

## Abstract

Folate and vitamin B_12_ are essential for growth. Our objective was to estimate their long-term effects on linear growth in North Indian children. This is a follow-up study of a factorial designed, double-blind, randomised, placebo-controlled trial in 1000 young children. Starting at 6–30 months of age, we gave folic acid (approximately 2 RDA), vitamin B_12_ (approximately 2 RDA), both vitamins or a placebo daily for 6 months. Six years after the end of supplementation, we measured height in 791 children. We used the plasma concentrations of cobalamin, folate and total homocysteine to estimate vitamin status. The effect of the interventions, the association between height-for-age z-scores (HAZ) and baseline vitamin status, and the interactions between supplementation and baseline status were estimated in multiple regression models. Mean (sd) age at follow-up was 7·4 (0·7) years (range 6 to 9 years). There was a small, non-significant effect of vitamin B_12_ on linear growth and no effect of folic acid. We observed a subgroup effect of vitamin B_12_ supplementation in those with plasma cobalamin concentration < 200 pmol/l (*P*_for interaction_ = 0·01). The effect of vitamin B_12_ supplementation in this group was 0·34 HAZ (95 % CI 0·11, 0·58). We found an association between cobalamin status and HAZ in children not given vitamin B_12_ (*P*_for interaction_ = 0·001). In this group, each doubling of the cobalamin concentration was associated with 0·26 (95 % CI 0·15, 0·38) higher HAZ. Suboptimal vitamin B_12_ status in early childhood seemingly limits linear growth in North Indian children.

Folate and vitamin B_12_ share metabolic pathways and are important for DNA and protein synthesis and therefore cell growth and differentiation^(1)^. Poor intake of either may accordingly be a contributing factor to poor growth worldwide. Many children in low- and middle-income countries have poor status of either or both of these nutrients^(2)^; the extent to which it has any consequences for growth is not known.

Deficiencies of folate and vitamin B_12_ are often part of general malnutrition and may contribute to poor growth in children in many low- and middle-income countries^(3–5)^. The main source of vitamin B_12_ is animal-derived foods, which are expensive and for cultural and religious reasons often not eaten at all^(2)^. Besides breast milk, the best sources of folates are dark green vegetables and legumes^(3)^. It is likely that these foods are inaccessible or only eaten in small amounts by many in poor populations.

Previously, we conducted a factorial design, double-blind, randomised controlled trial (RCT) of two RDA of vitamin B_12_ and/or folic acid supplementation in 1000 North Indian children aged 6 to 30 months. In the first and main phase of the study, we demonstrated that daily vitamin B_12_ supplementation for 6 months improved linear growth in children who were stunted, wasted or underweight at baseline^(6)^. Furthermore, vitamin B_12_ status at baseline was positively associated with linear growth in children who were *not* given vitamin B_12_ daily, but there were no such association in those who got vitamin B_12_. Other studies on the association between vitamin B_12_ status and growth and the effect of vitamin B_12_ supplementation on growth show conflicting results^(7,8)^.

The liver can store both folates and vitamin B_12_, and the effect of supplementation may persist long due to improved stores^(9,10)^. The objective of this follow-up study was to estimate if there was any effect of the vitamin B_12_ and/or folic acid supplementation during early childhood after 6 years (when the children were 6 to 9 years old), and the extent to which markers of these B-vitamins in early childhood were associated with or modified the effect of supplementation. The subgrouping variables were pre-specified and were based on the results from the paper reporting the effects on growth in the first phase of the RCT^(6)^.

## Materials and Methods

### Study settings, design and participants

We contacted children who previously participated in a factorial designed, randomised, double-blind, placebo-controlled trial on the effect of vitamin B_12_ and/or folic acid supplementation on childhood infections and growth in New Delhi, India, from January 2010 to September 2011^(11,12)^. Details on the baseline study procedures have previously been published^(11)^. Briefly, the children aged 6 to 30 months living in low- to middle-resource settings in New Delhi were randomised in blocks of 16 (in a 1:1:1:1 ratio) to receive either placebo, two RDA of vitamin B_12_, two RDA of folic acid or two RDA of both vitamins daily for 6 months. The randomisation list was generated in Stata by an individual not involved in the study. The allocation to the treatment groups was concealed, and the study participants, the study team and the involved scientists were blinded with regard to the group identity throughout the study period. The doses (approximately two RDA) for children aged above 12 months were 150 mcg of folic acid and 1·8 mcg of vitamin B_12_ (as cyanocobalamin). For children aged 12 months and below, the doses were 75 mcg and 0·9 mcg, respectively. The vitamins were delivered in a lipid-based nutritional supplement.

**Fig. 1.**
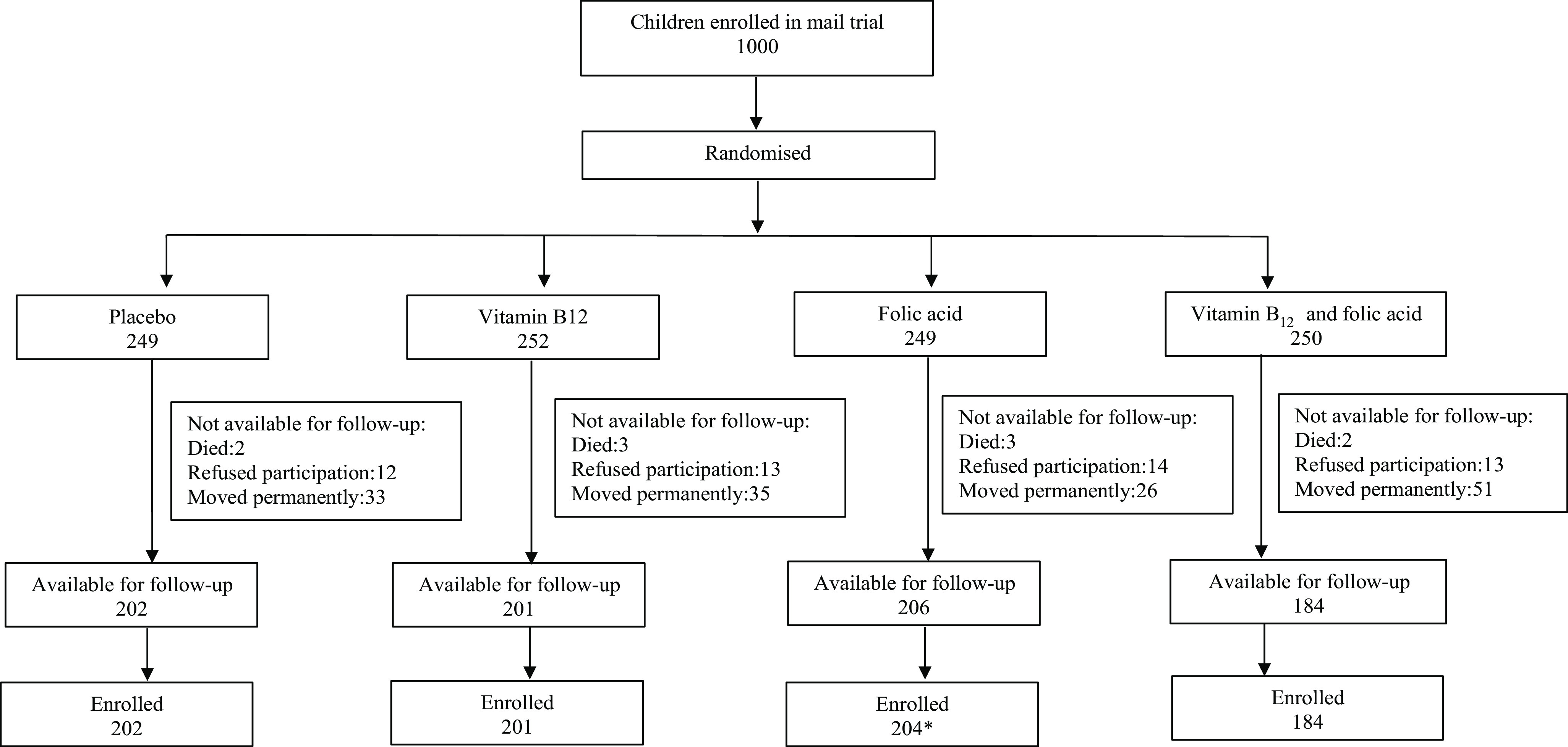
The participant flow in the study.

From September 2016, we approached the 1000 children from the baseline study and were able to get in contact with 798 of whom 791 consented to participate in this follow-up study (Fig. 1). All families were initially contacted by phone and invited to participate. A physical visit was made to the family’s address if no contact could be made. We requested the families who had moved out of the study area to come to the study clinic for a 1-d follow-up assessment. On the day of assessment, consent was taken from the children’s caregiver for participation in the study. Thereafter, we gathered information on socio-demography and the family situation through structured questionnaires.

The trial was first registered at www.clinicaltrials.gov as NCT00717730 in July 2008 and at www.ctri.nic.in as CTRI/2010/091/001090 in August 2010. The follow-up study was then registered as CTRI/2016/11/007494 in November 2016.

This study was conducted according to the guidelines laid down in the Declaration of Helsinki, and all procedures involving human subjects were approved by the Society for Applied Studies Ethics Review Committee [SAS/ERC/VitB12/2016] and Norwegian Regional Committee for Medical and Health Research Ethics (REK VEST) [2014/1359]. Written informed consent was obtained from the caregivers of the subjects.

### Assessments

Height was measured by Seca 213, to the nearest 0·1 cm twice in each child by a trained team of fieldworkers. Inter- and intra-observer standardisation exercises for anthropometric assessments were conducted before study initiation in the outcome ascertainment team, and these were repeated every 3 months throughout the study duration.

We obtained 3 ml of blood samples from all children at enrolment in a tube containing EDTA (Becton Dickinson). The plasma was centrifuged at approximately 1000 × *g* at room temperature for 10 min, separated, and transferred into storage vials and stored at −20°C until analysed. Microbiological assays estimated plasma concentration of vitamin B_12_ and folate^(13,14)^. Total plasma homocysteine (tHcy), a sensitive and functional marker for both vitamin B_12_ and folate deficiency, was analysed using commercial kits (Abbott Laboratories, Abbott Park)^(15)^.

### Statistical analyses

The original study included 1000 children. For this follow-up, we assumed that we would be able to re-enrol 800 children. With this sample size and assuming a sd of 1, we had 80 % power to identify an effect size of 0·2 height-for-age z-scores (HAZ) with a statistical significance of 0·05. We used the command ‘power twomeans’ in Stata for the sample size calculation. This calculation assumed no interaction between the two dimensions of the factorial RCT (folic acid and vitamin B_12_ supplementation).

Proportions, means (sd) or medians (interquartile range) were calculated for categorical and continuous variables by the four supplementation groups: vitamin B_12_, folic acid, vitamin B_12_ and folic acid and placebo for the baseline and follow-up characteristics of the children and their families. Children’s HAZ at follow-up was calculated using WHO growth standards^(16)^. The wealth of the families was determined by a wealth index created using principal component analysis based on assets owned by the household^(17)^. Using the scores from the principal component analysis, the population was divided into five equal wealth quintiles: poorest, very poor, poor, less poor and least poor.

Based on the RCT design, we estimated the effect of vitamin B_12_, folic acid and vitamin B_12_ and folic acid supplementation on linear growth (HAZ and stunting) at follow-up in crude and multivariable regression models. In the multivariable models, we adjusted for the wealth quintile, since this quintile was not evenly distributed between the intervention arms. We also merged the children receiving vitamin B_12_ into a ‘vitamin B_12_’ group (*n* 385) and compared this group with the children who did not receive vitamin B_12_ (‘placebo B_12_’ group, *n* 403). Similarly, we compared the children receiving folic acid (‘folic acid’ group, *n* 388) with the children who did not receive folic acid supplementation (‘placebo folic acid’ group, *n* 406). We measured the effects of supplementing vitamin B_12_ or folic acid in various pre-defined subgroups. We used the same subgroups (age at enrolment, sex, breast-feeding status, baseline anthropometric status, and cobalamin, folate and tHcy concentration at enrolment) as in the manuscript that reported the effects of vitamin B_12_, folic acid and vitamin B_12_ and folic acid on growth immediately after the end of supplementation^(6)^. We undertook these subgroup analyses unadjusted and adjusted for baseline age, wealth quintile, breast-feeding status, sex, as well as HAZ and weight-for-age z-scores. In these models, we also included interaction terms to measure whether the effects of vitamin B_12_ or folic acid were significantly different between the subgroups. We also measured the interaction between vitamin B_12_ and folic acid supplementation. Only one-way interactions were included in the statistical models.

In an observational (cohort) design, we examined the association between markers of vitamin B_12_ and folate (plasma cobalamin, folate and tHcy concentrations) at enrolment and HAZ and the proportion stunted at follow-up in multivariable regression models. To identify and adjust for confounders in these models, we used a method of purposeful selection of covariates^(18,19)^. The candidate variables were age and sex of the child, breast-feeding status at baseline, maternal and paternal years of schooling and wealth quintile. We also examined the interactions between vitamin B_12_ supplementation (vitamin B_12_ group) and cobalamin and tHcy concentrations, as well as the folic acid supplementation (folic acid group) and the folate and tHcy concentrations on linear growth (HAZ and stunting). The plasma concentrations of cobalamin, folate and tHcy were log (base 2)-transformed before used in the statistical analyses. We used generalised linear model with the Poisson distribution family and log link for dichotomous outcomes and the Gaussian distribution family and identity link for the continuous outcomes^(20)^. These statistical analyses were performed in Stata, version 16 (Stata corporation).

We also used generalised additive models in the statistical software R version 3.3.3 (The R Foundation for Statistical Computing) to explore non-linear associations between cobalamin, folate and tHcy concentrations at baseline and HAZ at follow-up adjusting for the same potential confounders as in the generalised linear models^(21)^. In these models, we present the association stratified by those who were and those who were not supplemented by vitamin B_12_. This stratified analysis was undertaken because of the interaction between vitamin B_12_ supplementation and vitamin B_12_ status.

## Results

Figure 1 shows the flow of the participants through the study. Of the 1000 children in the main study, 791 children consented to participate and were included in the follow-up study (Fig. 1). Demographic characteristics of the 791 participants at follow-up by the four intervention groups are shown in Table 1. Mean (sd) age at follow-up was 7·4 (0·7) years, ranging from 6 to 9 years.

**Table 1.** Demographic information of 791 North Indian children at baseline and follow-up (Number and percentages)

	Placebo		Vitamin B_12_		Folic acid		Vitamin B_12_ and folic acid	
	*n*	%	*n*	%	*n*	%	*n*	%
Number of participants	202		201		204		184	
Baseline study characteristics								
Child (6–30 months)								
Age baseline (months)								
Mean	16·3		15·9		16·4		16	
sd	7·0		6·9		7·2		7·0	
Boys	108	53·5 %	88	43·8 %	101	49·5 %	102	55·4 %
Still breastfed	159	78·7	164	82	160	78·8	149	81
Growth z-scores at baseline								
Weight-for-height z-scores (WHZ)								
Mean	–0·9		–0·9		–0·8		–0·9	
sd	0·9		1·0		1·0		0·9	
Height-for-age z-scores (HAZ)								
Mean	–1·8		–1·8		–1·8		–1·7	
sd	1·8		1·7		1·2		1·1	
Weight-for-age z-scores (WAZ)								
Mean	–1·7		–1·6		–1·5		–1·6	
sd	1·0		1·1		1·1		1·0	
Wasted (< –2 WHZ)	23	11·4	27	13·4	21	10·3	19	10·3
Stunted (< –2 HAZ)	79	39·1	76	37·8	80	39·2	61	33·1
Underweight (< –2 WAZ)	65	32·2	65	32·3	65	31·9	52	28·3
Biomarkers:								
Cobalamin (pmol/l)								
Mean	296·5		317		309·6		330·2	
sd	166·5		202·1		199·4		195·1	
Cobalamin < 200 pmol/l*	64	31·7	69	34·3	74	36·5	49	26·6
Folate (nmol/l)								
Mean	15·2		15·2		16. 1		15·4	
sd	12·9		13·9		14·6		14·2	
Folate < 7·5 nmol/l†	57	28·2	64	31·8	62	29·9	73	39·6
tHcy (μmol/l)								
Mean	14·8		13·2		13·6		13·4	
sd	8·6		7·2		7·5		6·9	
tHcy > 10 μmol/l‡	133	66·2	120	59·7	129	64·2	116	63·7
Child characteristics at follow-up (6–9 years)								
Age at follow-up (years)								
Mean	7·3		7·4		7·4		7·4	
sd	0·7		0·7		0·7		0·7	
Schooling								
No school	3	1·5	4	2	2	1	4	2·2
Hindi medium	74	37·4	64	32·2	52	25·7	52	28·3
English medium	121	61·1	131	65·8	148	73·3	128	69·6
Family characteristics at follow-up								
Annual family income (INR × 1000)								
Median	180		156		180		168	
IQR	120–336		120–288		120–300		120–288	
Mothers’ years of schooling								
No schooling	59	30·7	59	31·4	52	26·7	44	24·3
Primary (1–5 years)	24	12·5	24	12·8	27	13·8	26	14·4
Middle (6–12 years)	90	46·9	83	44·1	85	43·6	88	48·6
Higher (> 12 years)	19	9·9	22	11·7	31	15·9	23	12·7
Fathers’ years of schooling								
No schooling	23	11·4	18	8·9	20	9·8	20	10·9
Primary (1–5 years)	22	10·9	21	10·4	15	7·3	17	9·2
Middle (6–12 years)	131	64·8	141	70·1	136	66·7	119	64·7
Higher (> 12 years)	26	12·9	21	10·4	33	16·2	28	15·2
Wealth quintile								
Poorest	42	20·8	42	20·9	39	19·1	36	19·6
Very poor	43	21·3	51	25·4	34	16·7	30	16·3
Poor	37	18·3	42	20·9	35	17·2	44	23·9
Less poor	44	21·8	30	14·9	50	24·5	34	18·5
Least poor	36	17·8	36	17·9	46	22·5	40	21·7

* Data available in 790 children at follow-up.

† Data available in 791 at follow-up.

‡ Data available in 785 children at follow-up.

The mean (sd) and median (interquartile range) HAZ scores and the proportion of children stunted by intervention group are shown in Tables 2 and 3. Children who had been randomised to receive vitamin B_12_ were somewhat taller than the other children (mean difference of 0·12 HAZ (CI −0·01, 0·25), corresponding to a difference of 0·7 cm (CI −0·15, 1·57)); however, these differences were not statistically significant.

**Table 2.** HAZ scores in children of 6–9 years old according to intervention groups (Coefficient values and 95 % confidence intervals; mean values and standard deviations)

		HAZ score		Unadjusted		Adjusted	
Intervention group	*n*	Mean	sd	Coefficient	95 % CI*	Coefficient	95 % CI†
Three groups against the placebo							
Placebo	202	–1·05	1·1		Reference		Reference
Vitamin B_12_	201	–1·02	0·93	0·03	–0·16, 0·22	0·05	–0·12, 0·23
Folic acid	204	–1·08	0·97	–0·03	–0·22, 0·16	–0·08	–0·26, 0·09
Vitamin B_12_ and folic acid	184	–0·92	0·95	0·13	–0·07, 0·32	0·10	–0·08, 0·28
2 × 2 groups							
Placebo vitamin B_12_	403	–1·07	1·1		Reference		Reference
Vitamin B_12_	385	–0·97	0·94	0·09	–0·04, 0·22	0·12	–0·01, 0·25
Placebo folic acid	406	–1·03	1		Reference		Reference
Folic acid	388	–1·01	0·96	0·03	–0·10, 0·16	–0·03	–0·15, 0·10

HAZ, height-for-age z-scores.

* Generalised linear model with the Gaussian family and identity link.

† Adjusted for wealth quintiles.

**Table 3.** Proportion of children stunted at 6–9 years old according to intervention groups (Risk ratio and 95 % confidence intervals)

		Proportion stunted		Unadjusted		Adjusted	
Intervention group	*n*	*n*	%	RR	95 % CI*	RR	95 % CI†
Three groups against the placebo							
Placebo	202	33	16·3		Reference		Reference
Vitamin B_12_	201	28	13·9	0·85	0·52, 1·41	0·82	0·49, 1·35
Folic acid	204	37	18·1	1·11	0·69, 1·78	1·20	0·75, 1·92
Vitamin B_12_ and folic acid	184	23	12·5	0·76	0·44, 1·30	0·79	0·46, 1·35
2 × 2 groups							
Placebo vitamin B_12_	403	70	17·2		Reference		Reference
Vitamin B_12_	385	51	13·2	0·77	0·54, 1·10	0·73	0·51, 1·05
Placebo folic acid	406	60	15·1		Reference		Reference
Folic acid	388	60	15·5	1·02	0·71, 1·46	1·10	0·77, 1·58

* Generalised linear model with the Poisson family and log link.

† Adjusted for wealth quintiles.

**Fig. 2.**
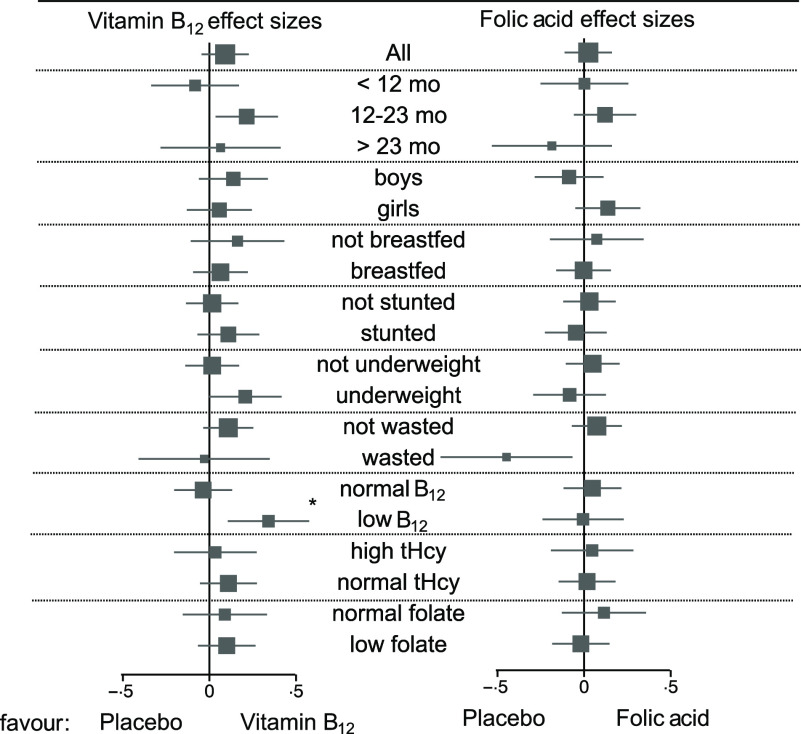
The effect of vitamin B_12_ or folic acid supplementation in early life on linear growth 6 years later by subgroups.

In the subgroup analyses, baseline cobalamin concentration modified the effect of vitamin B_12_ supplementation on growth (*P*_for interaction_ < 0·001). These analyses showed that there was an effect of vitamin B_12_ supplementation in those who had a baseline cobalamin concentration < 200 pmol/l. We also observed statistically significant effects in other subgroups (Fig. 2); however, the interaction term between these subgrouping variables was small and did not reach statistical significance.

The predictors for linear growth are shown in Table 4. The model shows that vitamin B_12_ supplementation modified the association between the cobalamin concentration at baseline and the HAZ score (*P*_for interaction_ < 0·001). Log cobalamin concentration at baseline predicted the HAZ score (regression coefficient 0·26; 95 % CI 0·15, 0·38) in the children who did not receive vitamin B_12_ supplementation, but not in the children who received vitamin B_12_ supplementation (–0·01; 95 % CI –0·13, 0·10). There was no such interaction between folic acid supplementation and folate concentration at baseline. There were no interactions between the ‘vitamin B_12_’ supplementation group and ‘folic acid’ supplementation group and tHcy concentration on HAZ (*P*_for interaction_ 0·230 and 0·953, respectively). The tHcy concentration at baseline was negatively associated with the HAZ scores (–0·15; 95 % CI –0·27, –0·01). The most saturated model with HAZ as outcome explained 20 % of the variability of the HAZ score at follow-up.

**Table 4.** Predictors for linear growth in children of 6–9 years old (Adjusted coefficient and 95 % confidence intervals, *n* 791)

	HAZ score			Stunting		
Variables	Adjusted coefficient	95 % CI	*P*	Adjusted RR	95 % CI	*P*
Ever breastfed (ref. never breastfed)	1·01	0·39, 1·63	0·001†	0·26	0·10, 0·65	0·004†
Mother’s years of education	0·02	0·006, 0·04	0·006†	0·95	0·90, 0·99	0·027†
Wealth quintile						
Poorest		Reference			Reference	
Very poor	0·21	0·01, 0·42	0·036†	0·68	0·43, 1·10	0·071
Poor	0·21	0·002, 0·41	0·048†	0·69	0·41, 1·14	0·146
Less poor	0·45	0·24, 0·66	< 0·001†	0·41	0·22, 0·77	0·006†
Least poor	0·85	0·61, 1·10	< 0·001†	0·20	0·10, 0·55	0·002†
Total plasma homocysteine concentration*	–0·15	–0·27, −0·01	0·025†	1·31	0·92, 1·86	0·139
‘Vitamin B_12_’ group and cobalamin concentration interaction	–0·28	–0·44, −0·12	0·001†	1·78	1·15, 2·74	0·009†
Cobalamin concentration† in the ‘placebo B_12_’ group	0·26	0·15, 0·38	< 0·001†	0·70	0·53, 0·93	0·014†
Cobalamin concentration† in the ‘vitamin B_12_’ group	–0·01	–0·13, 0·10	0·822	1·25	0·90, 1·74	0·176

HAZ, height-for-age z-scores.

* log (base 2)-transformed value.

† *P* < 0.05.

The association between cobalamin concentration at baseline and stunting was modified by vitamin B_12_ supplementation (*P*_for interaction_ = 0·004) (Table 4). In this analysis, the log-transformed cobalamin concentration at baseline was associated with stunting in the children who did not receive vitamin B_12_ supplementation (Relative Risk (RR) 0·70; 95 % CI 0·53, 0·93), but not in the children who did (RR 1·25; 95 % CI 0·90, 1·74). We did not see such interaction between the ‘folic acid’ supplementation group and folate status at baseline. Breast-feeding and wealth quintiles were found to be predictors for both HAZ and stunting at follow-up (Table 4).

**Fig. 3.**
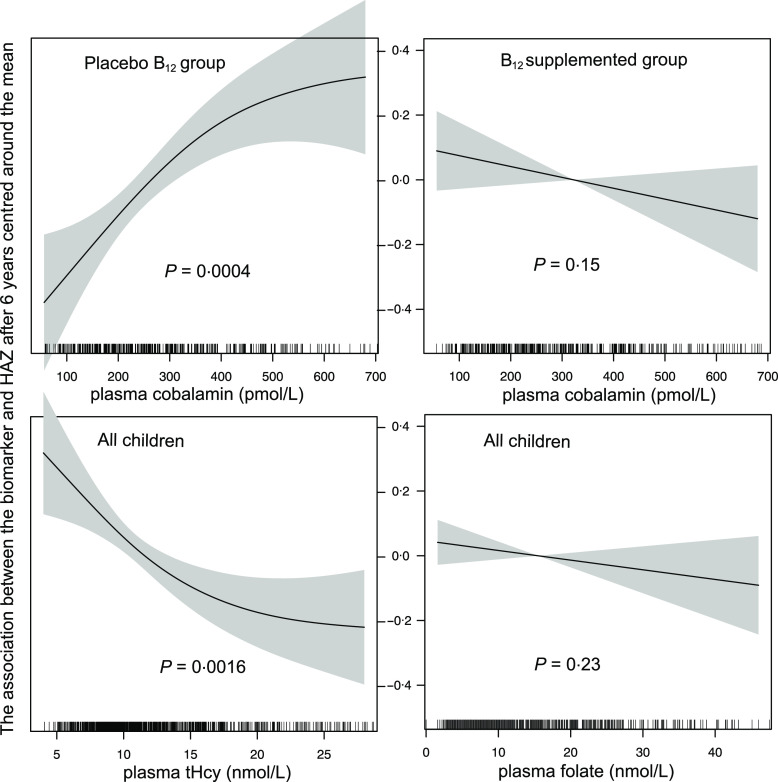
The association between plasma cobalamin, folate and tHcy during early childhood and linear growth 6 years later. The y-axis denotes the HAZ scores centred around the mean, and the x-axis denotes baseline plasma cobalamin, tHcy and folate concentrations. The graphs were constructed using generalised additive models in R, and the solid line depicts the association of plasma cobalamin, folate and tHcy during early childhood and HAZ 6 years later. The shaded area spans the 95 % CI of these associations. HAZ, height-for-age z-scores.

The association between plasma cobalamin, folate and tHcy concentrations at baseline and HAZ scores at follow-up is depicted in Fig. 3. The figures show that HAZ scores increase with increasing log cobalamin concentration in the children who did not receive vitamin B_12_ supplementation, but not in the children who received vitamin B_12_. There was no association between log folate status at baseline and HAZ score at follow-up, but the HAZ scores decreased with increasing log tHcy levels at baseline. The generalised additive model plots did not reveal any non-linear associations.

## Discussion

In the original trial, reporting the short-term effects, we found that 6 months of vitamin B_12_ supplementation resulted in a modest but statistically significant effect on weight-for-age z-scores^(6)^. We also found an effect of vitamin B_12_ supplementation on linear growth in subgroups of children who were stunted, underweight or wasted at baseline. In the current follow-up study, we found that vitamin B_12_ supplementation resulted in a small (statistically insignificant) overall effect on linear growth after 6 years. There was also a slight reduction in the risk of stunting in the vitamin B_12_ group. The 95% CI, however, of this measured effect were consistent with a 49 % reduction to a 5 % increase in the risk of stunting compared with placebo.

The subgroup analyses found that vitamin B_12_ status at baseline modified the effect of vitamin B_12_ supplementation on linear growth, demonstrating a substantial effect in children with baseline subclinical vitamin B_12_ deficiency but not in those with adequate status. Similarly, when using data in a prospective cohort design, the association between baseline cobalamin concentration was modified by vitamin B_12_ supplementation. The association between baseline vitamin B_12_ status (pre-enrolment plasma cobalamin concentration) and linear growth was observed only in children who were not supplemented with vitamin B_12_. In the children who did not receive vitamin B_12_ supplement (placebo B_12_ group), each unit increment in log (base2) plasma cobalamin concentration (i.e. a doubling of the concentration) was associated with a 0·26 increase in HAZ score and a 30 % reduction in the risk of being stunted after 6 years. This interaction between vitamin B_12_ status and supplementation suggests a causal effect of vitamin B_12_ supplementation on growth. We believe that we can suggest causality from this observational finding, because the effect modifier (i.e. supplementation) is a variable that has been randomised and at the same time affects vitamin B_12_ status.

Vitamin B_12_ is involved in two biochemical reactions in humans^(22)^. One of these, the methionine cycle is also dependent on an adequate supply of folate for the remethylation of homocysteine to methionine. Disruption of this cycle increases homocysteine and will affect gene regulation, DNA synthesis and, subsequently, growth^(22)^. Incomplete methylation can also limit linear growth through senescence of the resting zone chondrocytes of the growth plates^(23)^.

The results from our analyses indicate that folate is not a growth-limiting nutrient in this population. This is also supported by the fact that only plasma cobalamin, and not folate concentration, was negatively associated with plasma tHcy concentration at baseline. In the other metabolic pathway where vitamin B_12_ takes part, the B-vitamin acts as an enzymic cofactor for methylmalonyl CoA mutase, which is involved in the catabolism of certain fats and amino acids that could be important for growth. This reaction is not linked to folate metabolism, and an effect of vitamin B_12_ through this pathway could also explain the lack of interaction between folic acid and vitamin B_12_ supplementation.

Our findings from the observational design are in line with a recently published study from Nepal where vitamin B_12_ status estimated by cobalamin, methylmalonic acid and tHcy concentration in infancy was associated with linear growth 5 years later^(7)^. In this cohort study, infant status and maternal vitamin B_12_ intake and status during lactation were positively associated with growth when the children were around 5 years of age. However, the results from another study, also from Nepal, are at odds with the findings from the RCT design-results of this study^(8)^. In this latter study, 2 µg of cyanocobalamin daily from infancy and for 1 year resulted in a metabolic response reflecting improved vitamin B_12_ status but had no effect on growth. The discrepancy between these RCTs can be explained by the many differences between the study populations. The daily dose and age of enrolment were reasonably similar between the studies. However, the study in Nepal restricted enrolments to children who were mildly stunted (< –1 HAZ upon enrolment). By focusing on a marginalised group of children from a relatively poor community, there could have been other health or nutritional factors that limited the response of supplementation on growth. It should be noted that both RCTs demonstrated a beneficial effect of vitamin B_12_ supplementation on the metabolic profile, which, in turn, may have implications for health. Similar to our findings, other studies from low- and middle-income countries have showed that mothers’ years of education, socio-economic and breast-feeding status were independent predictors of stunting^(24,25)^. This is an important reminder of the many factors that are important for healthy growth, all of which could modify the effect of supplementation of single nutrients.

There are some limitations to the study. First, the dosage might have been too small and the duration of supplementation too short. Second, the absorption of the vitamins may have been interfered by bacterial overgrowth^(10)^. Third, impaired growth can also be caused by deficiencies of other growth-limiting nutrients, repeated infections and poor dietary quality^(26)^. Fourth, no dietary assessments were done participants and parents. Some of these limitations may have underestimated the potential effect by optimising vitamin B_12_ or folate status. In other words, we might have overlooked important effects because the status was not improved sufficiently or because of other factors that affected growth. However, we do not believe that these factors could have attenuated our effect estimates substantially because we found a large effect of the supplementation of vitamin B_12_ in those with inadequate status. Lastly, the 20 % attrition rate is a limitation and could be a source of bias. However, there were no major differences between the children who were included in the follow-up and those who were not on the relevant demographic characteristics.

Successful inclusion of 80 % of the children from the baseline trial after approximately 6 years, high quality and robust assessment of growth, and the availability of different plasma nutrient biomarker concentrations are the strengths of the study. Another strength of the study is the randomised, double-blind design of the original trial enabling causal inferences from the observed results.

The results of this study could have important public health implications. Individuals who do not consume animal foods may benefit from vitamin B_12_-fortified foods or oral vitamin B_12_ supplements.

### Conclusion

Our findings show that vitamin B_12_ deficiency limits growth and that vitamin B_12_ supplementation improves growth and reduces the risk of stunting in North Indian children with subclinical vitamin B_12_ deficiency. Being a growth-limiting nutrient in young children in New Delhi, vitamin B_12_ supplementation should be considered.
